# Improvement of *n*-caproic acid production with *Ruminococcaceae* bacterium CPB6: selection of electron acceptors and carbon sources and optimization of the culture medium

**DOI:** 10.1186/s12934-018-0946-3

**Published:** 2018-06-25

**Authors:** Han Wang, Xiangzhen Li, Yi Wang, Yong Tao, Shaowen Lu, Xiaoyu Zhu, Daping Li

**Affiliations:** 1 Key Laboratory of Environmental and Applied Microbiology, Chengdu Institute of Biology, Chinese Academy of Sciences, Chengdu, 610041 China; 2 Environmental Microbiology Key Laboratory of Sichuan Province, Chengdu Institute of Biology, Chinese Academy of Sciences, Chengdu, 610041 China; 3 University of Chinese Academy of Sciences, Beijing, 100049 People’s Republic of China; 4 Department of Biosystems Engineering, Auburn University, Auburn, AL 36849 USA

**Keywords:** Medium-chain carboxylic acid, *n*-Caproic acid (CA), Electron acceptor (EA), Chain elongation, *Ruminococcaceae* bacterium CPB6

## Abstract

**Background:**

Global energy and resource shortages make it necessary to quest for renewable resources. *n-*Caproic acid (CA) production based on carboxylate platform by anaerobic fermentation is booming. Recently, a novel *Ruminococcaceae* bacterium CPB6 is shown to be a potential biotransformation factory for CA production from lactate-containing wastewater. However, little is known about the effects of different electron acceptors (EAs) on the fermentative products of strain CPB6, as well as the optimum medium for CA production.

**Results:**

In this study, batch experiments were performed to investigate the fermentative products of strain CPB6 in a lactate medium supplemented with different EAs and sugars. Supplementation of acetate, butyrate and sucrose dramatically increased cell growth and CA production. The addition of propionate or pentanoate resulted in the production of C5 or C7 carboxylic acid, respectively. Further, a Box–Behnken experiment was conducted to optimize the culture medium for CA production. The result indicated that a medium containing 13.30 g/L sucrose, 22.35 g/L lactate and 16.48 g/L butyrate supported high-titer CA production (16.73 g/L) with a maximum productivity of 6.50 g/L/day.

**Conclusions:**

This study demonstrated that strain CPB6 could produce C6–C7 carboxylic acids from lactate (as electron donor) with C2–C5 short-chain carboxylic acids (as EAs), but CA (C6 carboxylic acid) was the most major and potential product. Butyrate and sucrose were the most significant EA and carbon source respectively for CA production from lactate by strain CPB6. High titer of CA can be produced from a synthetic substrate containing sucrose, lactate and butyrate. The work provided significant implications for improving CA production in industry-scale.
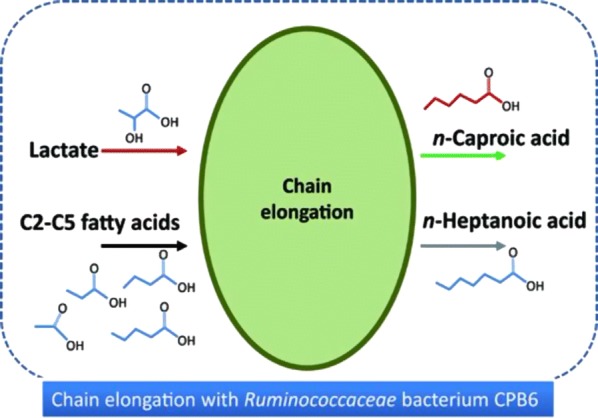

**Electronic supplementary material:**

The online version of this article (10.1186/s12934-018-0946-3) contains supplementary material, which is available to authorized users.

## Background

Recently, the renewable chemical production via the carboxylate platform garners a lot of attentions [1, 2]. In the carboxylate platform, complex waste is firstly converted into intermediate short-chain carboxylic acids (SCCAs, C2–C5) by anaerobic microbiomes, and then SCCAs are further chain-elongated to medium-chain carboxylic acids (MCCAs, C6–C8) via the reverse β-oxidation pathway [3, 4].

*n*-Caproic acid (CA, C6), a 6-carbon-chain carboxylic acid, is an important industrial chemical for several industrial applications including antimicrobial agent [5], fodder annexing agent [6, 7], rubbers [1] and precursor of aviation fuels [8, 9]. CA is one of the most potential products in the carboxylate platform because it has already been shown to be produced by microorganisms with high productivity from either ethanol and acetate [10–12], or beer wastewater with undiluted ethanol [3], or syngas fermentation including acetate and dilute ethanol [13]. The well-known pathway for CA production is reverse β-oxidation occurring with ethanol as electron donor (ED) and acetate as electron acceptor (EA) [2–4, 9]. Ethanol is a most efficient reduced substrate (as ED) because its biooxidation can provide energy (ATP) and reducing equivalents (NADH), and acetyl-CoA to drive the reverse β-oxidation for chain elongation. Several compounds, such as acetate, propionate, butyrate, as well as succinate and malate, have been reported to be EAs for MCCAs production [10, 14]. Lactate has also been described to generate the acetyl-CoA to provide the two carbon atoms for the acetate to *n*-butyrate elongation via reverse β-oxidation, in which the oxidation of lactate to pyruvate produces NADH and the conversion of pyruvate to acetyl-CoA with ATP generation. However formerly, propionate and butyrate are the major products, and few caproate is produced from lactate [4, 15]. Recently, a series of studies showed that lactate (as ED) can be efficiently converted into CA with reactor microbiomes [16, 17]. More recently, a novel *Ruminococcaceae* bacterium CPB6 is shown to be capable of producing high concentration CA from lactate-containing wastewater [18]. Therefore, lactate has been considered as another effective substrate for CA production.

Lactate is an important intermediate during anaerobic oxidation of the carbohydrates, constituting a high proportion soluble chemical oxygen demand (COD) in organic waste streams [16, 19–21]. The conversion of lactate to CA provides a potential approach to produce high added-value product from organic wastes. So far, only two pure cultures (*Megasphaera elsdenii* and *Ruminococcaceae* bacterium CPB6) are reported to produce CA from lactate. Nevertheless, the fact that low titer CA production (< 0.5 g/L) from *M. elsdenii* using lactate indicates that CA is only a negligible byproduct [22]. By contrast, strain CPB6 can produce much higher-titer of CA (16.6 g/L) from lactate-containing wastewater than *M. elsdenii* [18]. The strain provides us an ideal model to study regulation mechanisms and pathways of CA production from lactate.

In recent study, we have preliminary investigated the optimum pH and temperature for the growth of strain CPB6 that can produce CA from lactate [18]. Moreover, the whole genome of the strain has been sequenced, and a sets of genes involved in the fatty acid biosynthesis via acyl carrier protein (ACP) and coenzyme A (CoA) as well as lactate oxidation/reduction pathways in the genome are identified [23]. However, the route of lactate conversion into CA still remains largely unexplored, e.g., what is the suitable EA, and what is the optimum medium for CA production, as well as whether this process is similar to the ethanol-based chain elongation pathway. In this study, we investigated the effects of different EAs on MCCAs production from lactate, and selected the optimum EA and carbon source on cell growth and CA production. On this basis, we further conducted optimization of media for CA production by using Box–Behnken experimental design for efficient CA production.

## Results and discussion

### Fermentation products of CPB6 with different EAs

**Fig. 1:**
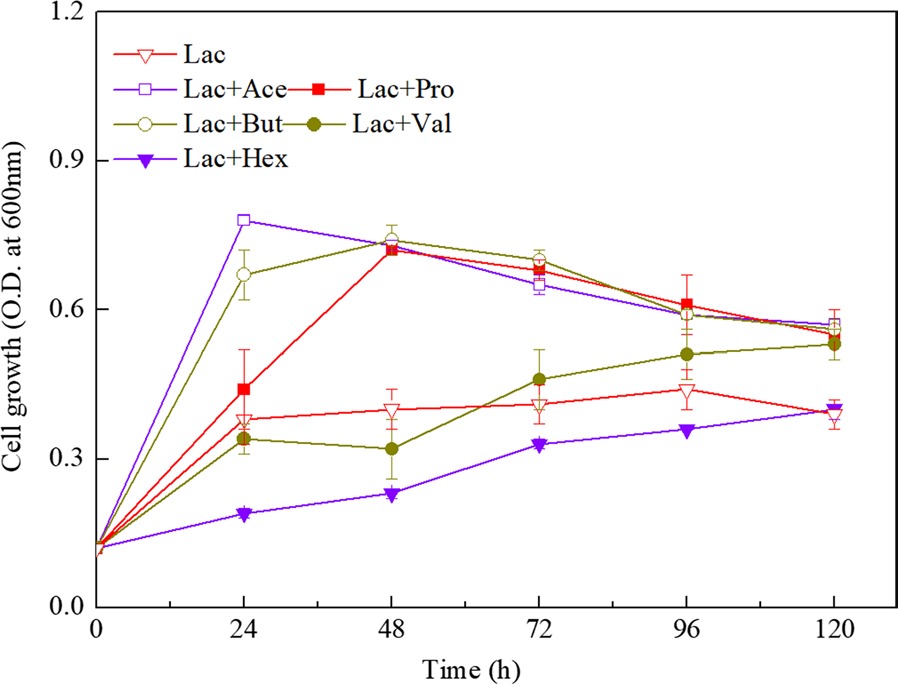
The growth of strain CPB6 in lactate medium supplemented with C2–C6 electron acceptors. All data were presented as means ± standard deviations (n = 3)

As shown in Fig. 1, strain CPB6 grew poorly (maximum optical density of the sample measured at a wavelength of 600 nm (OD_600_ = 0.4) in the lactate (sole energy substrate) medium without EA. Only the low concentration of CA (1.96 g/L) was produced as major product after 5 days of culture. When either 100 mM acetate (C2) or butyrate (C4) was supplemented to the lactate-medium, cell biomass increased remarkably, and OD_600_ reached 0.8 and 0.6 respectively after 1 day of cultivation (Fig. 1). After 5 days of culture, CA titers were 4.62 and 8.07 g/L respectively (Table 1), increasing 2.35 and 4.12-fold than the control. When acetate or butyrate was replaced by 100 mM propionate (C3) in the lactate-medium, cell growth was nearly consistent with that of butyrate addition (Fig. 1). Pentanoic acid (C5, 4.38 g/L) instead of CA (C6, 1.76 g/L) was the major product, and 0.68 g/L *n*-heptanoic acid (C7) was detected in the 5-day culture (Table 1). The growth delay of CPB6 was observed when pentanoate (C5) and caproate (C6) were added to the medium (Fig. 1). After 5-day culture, approximate 2 g/L *n*-heptanoic acid (C7) was observed in the medium supplemented with pentanoate (Table 1). However, no *n*-octanoic acid was detected when CA was EA. It was different from *Megasphaera* sp. MH, another CA-producing bacteria, which is recently reported to produce various MCCAs from different EAs, e.g., it produces CA (9.7 g/L) as the major product when acetate and butyrate are EAs, while *n*-octanoic acid (C8) is the main product when acetate and caproate are used as EAs [24]. There was no *n*-octanoic acid production by CPB6 in all the groups, implying that strain CPB6 was not suitable to produce MCCAs longer than C7.

**Table 1:** The fermentation products of CPB6 with different electron acceptors (EAs)

Groups	Lactate consumption (g/L)	Fermentation products (g/L)^a^						
		Acetic acid	Propionic acid	Butyric acid	Pentanoic acid	Caproic acid	Heptanoic acid	Octanoic acid
Without EA	5.49	ND	ND	ND	ND	1.96 ± 0.10	ND	ND
With EA								
Ace	11.66	5.49 ± 0.22^b^	ND	1.12 ± 0.06	ND	4.62 ± 0.17	ND	ND
Pro	11.76	1.41 ± 0.03	3.22 ± 0.09^b^	0.87 ± 0.03	4.38 ± 0.15	1.76 ± 0.16	0.68 ± 0.07	ND
But	11.86	1.72 ± 0.07	ND	4.05 ± 0.13^b^	ND	8.07 ± 0.18	ND	ND
Pen	7.90	ND	ND	ND	6.49 ± 0.31^b^	1.78 ± 0.21	2.09 ± 0.31	ND
Cap	5.31	ND	ND	ND	ND	9.00 ± 0.15^b^	ND	ND

*ND* not detected

^a^ The data represents the average of triplicate determinations after 3-day cultivation. The fermentation medium contains 12 g/L lactate with 100 mM different electron acceptors including acetate, propionate, butyrate and pentanoate except caproate (50 mM)

^b^ The values are undefined as products or unspent substrates

The fact that exogenous acetate increases cell growth is observed in other chain-elongating bacteria, such as *C. kluyveri* that produces CA from ethanol [10], *Megasphaera* sp. MH that produces CA from fructose [24], *Clostridium* sp. BS-1 that produces CA from d-galactitol [25], and *Clostridium* sp. BPY5 that produces butyrate from lactate [15]. This is possibly because the electron flows inside the cell are changed by the electron equivalent from acetate (8 electron equivalents per mole), leading to the increase of cell growth [26]. Compared to the control group (without EAs), exogenous electron equivalent might result in more end-products (e.g., CA or butyrate) in the acetate addition group. Likewise, exogenous butyrate and propionate might play the similar roles in increasing cell growth and end-products. Notably, CA production with butyrate as EA was significantly higher than that with acetate as EA, likely because exogenous butyrate provided more electron equivalent than acetate (8 vs 20 electron equivalents per mol).

**Fig. 2:**
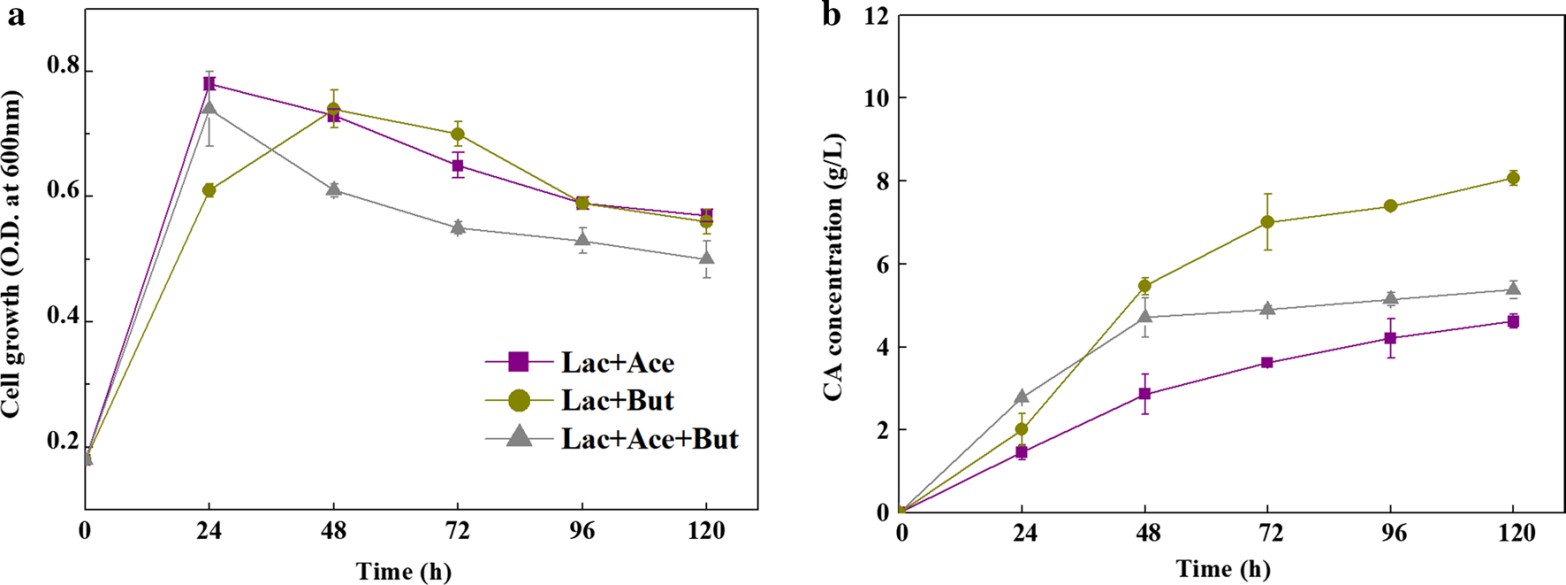
The growth (**a**) of strain CPB6 and *n*-caproic acid production (**b**) in a 5-day co-fermentation with acetate and butyrate. All data were presented as means ± standard deviations (n = 3)

CA production rate was quicker in the butyrate added group than the acetate added group (1.614 g/L/day vs 0.93 g/L/day, Table 1). One possible reason is that in the acetate added group, acetyl-CoA (that was derived from lactate) and acetate must firstly be elongated to butyryl-CoA [1, 4], and then the butyryl-CoA with another acetyl-CoA (that was derived from lactate) was elongated to CA via chain elongation pathway. In butyrate added group, butyrate with acetyl-CoA (that was derived from lactate) could be directly converted to CA via the reverse β-oxidation pathway. In addition, when both acetate and butyrate were added as cosubstrates to the lactate medium, the OD_600_ during exponential phase was almost in line with the fermentation when acetate or butyrate was added individually (Fig. 2a). The CA production with cosubstrates supplemented was higher than that with acetate addition but lower than that with butyrate addition (Fig. 2b). This result was different from that of Jeon et al. [24], in which *Megasphaera* sp. MH produced more CA in cosubstrates (acetate and butyrate) than that in the sole substrate of acetate or butyrate. The reason for this warrants further study.

The addition of propionate led to the production of pentanoic acid (C5), indicating propionate elongation was linked with coupling a first propionyl-CoA molecule (that was derived from propionate) to a second acetyl-CoA molecule (that was derived from lactate) in the reverse β-oxidation cycle. Small amounts of CA might be resulted from the conversion of lactate to CA, which has been proved in the previous study [18]. The growth delay of CPB6 in the pentanoate or caproate added group might be attributed to the toxicity of C5–C6 fatty acids to microorganism in low initial pH (pH = 5.5) in this study. This phenomenon is also observed in *Megasphaera elsdenii* [27] and *Megasphaera* sp. MH [24].

### Effect of sucrose on cell growth and CA production

**Fig. 3:**
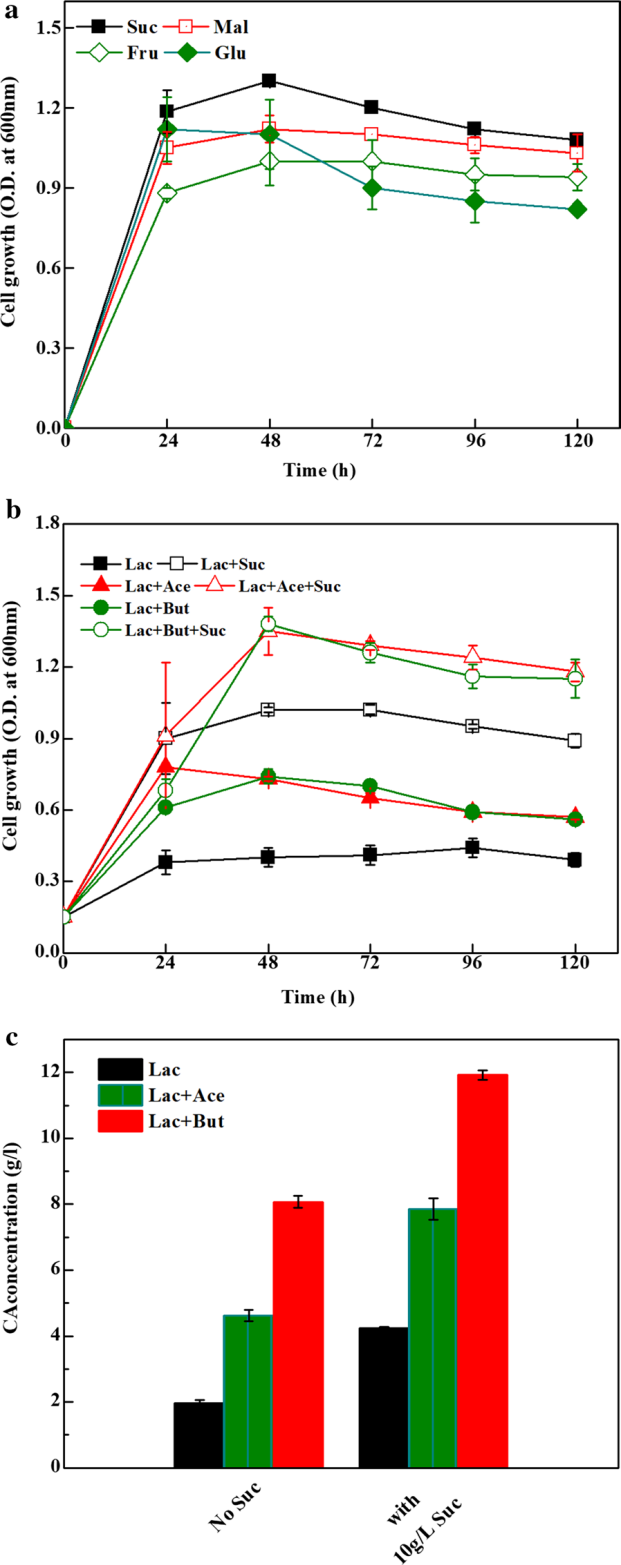
The effect of sugars on cell growth (**a**); and the effect of sucrose on cell growth (**b**); and CA production in lactate (11.7 g/L) medium supplemented without EA and with 100 mM acetate or butyrate in a 5-day fermentation (**c**). All data were presented as means ± standard deviations (n = 3)

The growth of strain CPB6 on four carbon sources (sucrose, maltase, fructose, and glucose, final concentration 10 g/L) were shown in Fig. 3. The results showed that sucrose was superior to other carbohydrates for cell growth (Fig. 3a). Therefore, the effect of sucrose on CA production was further investigated. As shown in Fig. 3b, sucrose supplementation improved remarkably cell growth in lactate-medium without EA (increasing OD_600_ from 0.4 to 1.2) and with EA (e.g., acetate or butyrate, OD_600_ from 0.8 to 1.4). Meanwhile, compared to the control (without adding EA), the addition of sucrose enhanced CA production from 1.96 to 4.25 g/L. Likewise, in the cultures with acetate or butyrate as EAs, sucrose supplementation led to a significant increase of CA production (from 4.62 to 7.85 g/L, and 8.07 to 11.92 g/L, respectively) (Fig. 3c). These results demonstrated that CA production could be improved dramatically when sucrose was added to lactate-medium with- or without EAs. Furthermore, the stoichiometric balances showed that the addition of sucrose reduced lactate consumption (33–41%) in 1 mol of CA production, e.g., the lactate consumption dropped from 3.6 to 2.22 in the control group. In acetate or butyrate added group, the required lactate for 1 mol CA production also decreased from 3.25 to 1.91, and from 1.88 to 1.26, respectively (Table 2). This might be because more acetyl-CoA from the glycolysis of sucrose entered chain elongation for CA production. In this study, sucrose showed positive effects on cell growth and CA production, indicating that sucrose can be used as an economic carbon source for CA production from lactate by strain CPB6 in large-scale production.

**Table 2:** Stoichiometric balances for the fermentation using strain CPB6 grown on different substrates

Substrates	1 mol caproate produced from^a^		
	Acetate	Butyrate	Lactate
Lactate	ND	ND	− 3.6
Lactate + sucrose	ND	ND	− 2.22
Lactate + acetate	− 0.82	0.33	− 3.25
Lactate + acetate + sucrose	− 0.37	0.29	− 1.91
Lactate + butyrate	0.42	− 0.75	− 1.88
Lactate + butyrate + sucrose	0.26	− 0.57	− 1.26

*ND* not detected

^a^ Unit is moles

“–” means net consumption of substrate

### Optimization of culture medium for CA production

In this study, a full factorial experiment of yeast extract, tryptone, sucrose and butyrate was conducted to screen significant factors affecting CA production, (see Additional file 1: Tables S1 and S2). The influence of yeast extract and tryptone on CA production was weak and was excluded from further evaluation. The final concentration of yeast extract and tryptone were set at 5 g/L in each experiment. Afterwards, according to the results of the full factorial experiment design, a steepest ascent experiment (see Additional file 1: Table S3) was performed to determine the concentration ranges of sucrose, butyrate and lactate for the following Box–Behnken experimental design. Higher CA production was observed in the run 2 and run 3, so the average of values of run 2 and run 3 was used as the center point for the Box–Behnken (Table 3). The ranges of concentration of sucrose, lactate and butyrate were 5–20 g/L, 18–26 g/L and 12–18 g/L, respectively. A quadratic model was obtained by multiple regression analysis of five replicates at the center point, and 12 experiments were designed by Box–Behnken model with above three variables. The equation for predicting CA production was as follows:

1 $$\begin{aligned} {\text{Y}} &= 1 6.0 3 { } + \, 0. 3 4 {\text{X}}_{ 1} + \, 0. 70{\text{X}}_{ 2} + \, 0. 3 9 {\text{ X}}_{ 3} - \\& \quad \;0. 1 1 {\text{ X}}_{ 1} {\text{X}}_{ 2} {-} \, 0. 30{\text{ X}}_{ 1} {\text{X}}_{ 3} {-} \, 0. 1 7 {\text{X}}_{ 2} {\text{X}}_{ 3} \\&\quad \; - \, 0. 7 2 {\text{ X}}_{1}^{2} {-} \, 0. 7 7 {\text{ X}}_{2}^{2} {-} \, 0. 2 9 {\text{ X}}_{3}^{2} \hfill \\ \end{aligned}$$

where Y is the predicted response (CA concentration), and X_1_, X_2_ and X_3_ are the coded values of the sucrose, lactate and butyrate, respectively.

**Table 3:** Levels of the variables for the Box–Behnken experimental design

Variables	Symbol	Coded levels		
		− 1	0	+ 1
Sucrose (g/L)	X1	5	12.5	20
Lactate (g/L)	X2	18	22	26
Butyrate (g/L)	X3	12	15	18

The Box–Behnken experimental design and results of all factor levels were displayed in Table 4. An analysis of variance (ANOVA) as performed for evaluating the appropriateness of the quadratic regression mode (see Additional file 1: Table S4).

**Table 4:** Box–Behnken experimental design matrix and results

Run	X1^a^	X2^a^	X3^a^	Sucrose	Lactate	Butyrate	*n*-Caproic acid (g/L)
1	0	− 1	1	12.5	14	18	14.71
2	0	1	1	12.5	26	18	15.78
3	− 1	1	0	5	26	15	15.00
4	1	0	1	20	20	18	15.55
5	− 1	0	− 1	5	20	12	13.89
6	0	0	0	12.5	20	15	16.01
7	1	1	0	20	26	15	15.47
8	0	0	0	12.5	20	15	16.17
9	0	1	− 1	12.5	26	12	15.55
10	0	0	0	12.5	20	15	16.23
11	0	− 1	− 1	12.5	14	12	13.8
12	0	0	0	12.5	20	15	15.89
13	1	0	− 1	20	20	12	15.16
14	− 1	− 1	0	5	14	15	13.39
15	0	0	0	12.5	20	15	15.84
16	− 1	0	1	5	20	18	15.46

^a^ X1: sucrose, X2: lactate, X3: butyrate

The F-value of 47.28 and *P* value < 0.0001 indicated that the model was significant. The R^2^ value was 98.38% and the R^2^_adj_ value was 96.30%, ensuring the reliability of the model in this study. The P value of “lack of fit” was 0.49, showing a good prediction of the mode fit. The results of ANOVA indicted that all the three factors had significant effects on CA production (*P *≤ 0.05).

**Fig. 4:**
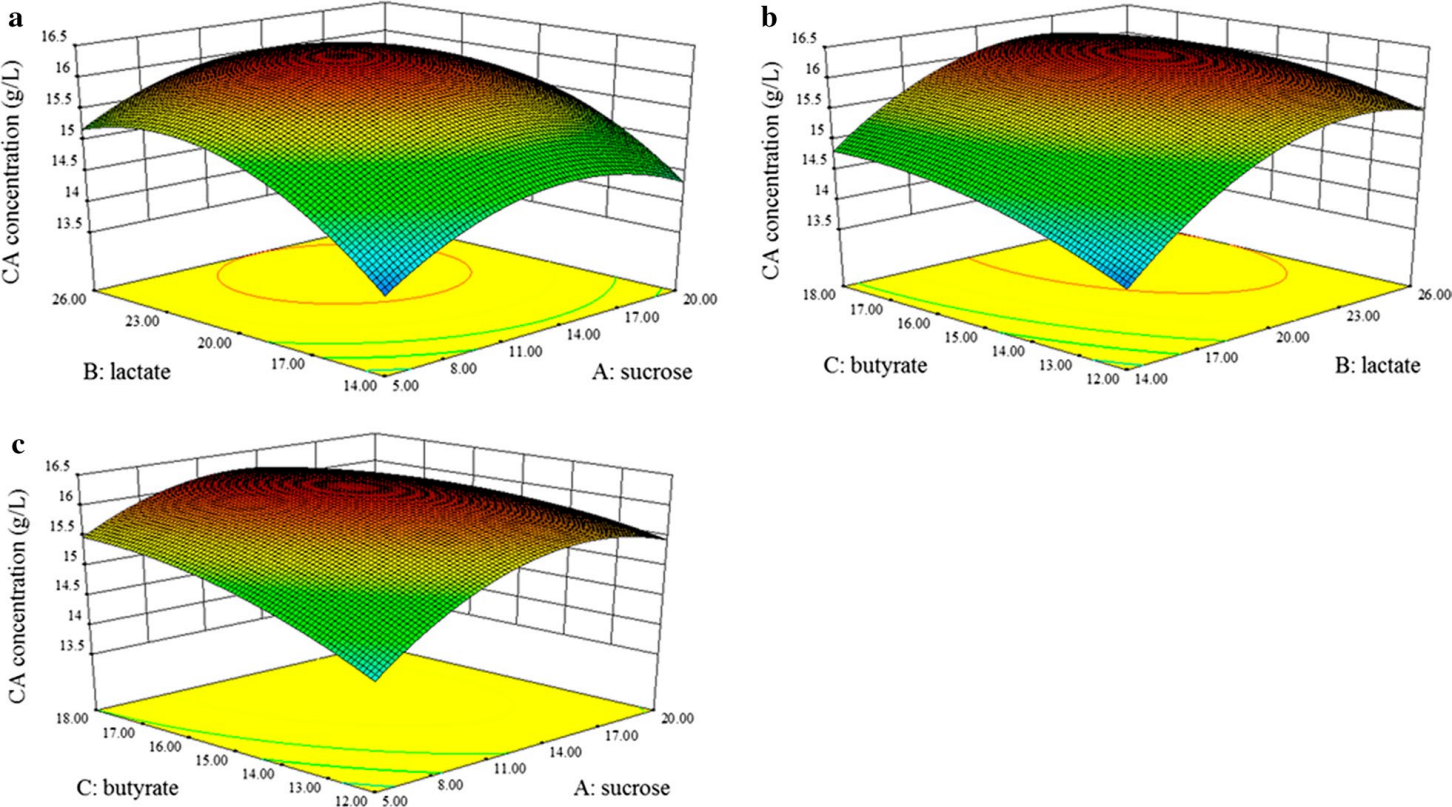
The response surface plot showing a function of sucrose, lactate, butyrate on CA production. **a** The interaction of sucrose and lactate on CA production with butyrate at 16.2 g/L; **b** the interaction of butyrate and lactate on CA production with sucrose at 13.3 g/L; **c** the interaction of sucrose and butyrate on CA production with lactate at 22.35 g/L. All data were presented as means ± standard deviations (n = 3)

**Fig. 5:**
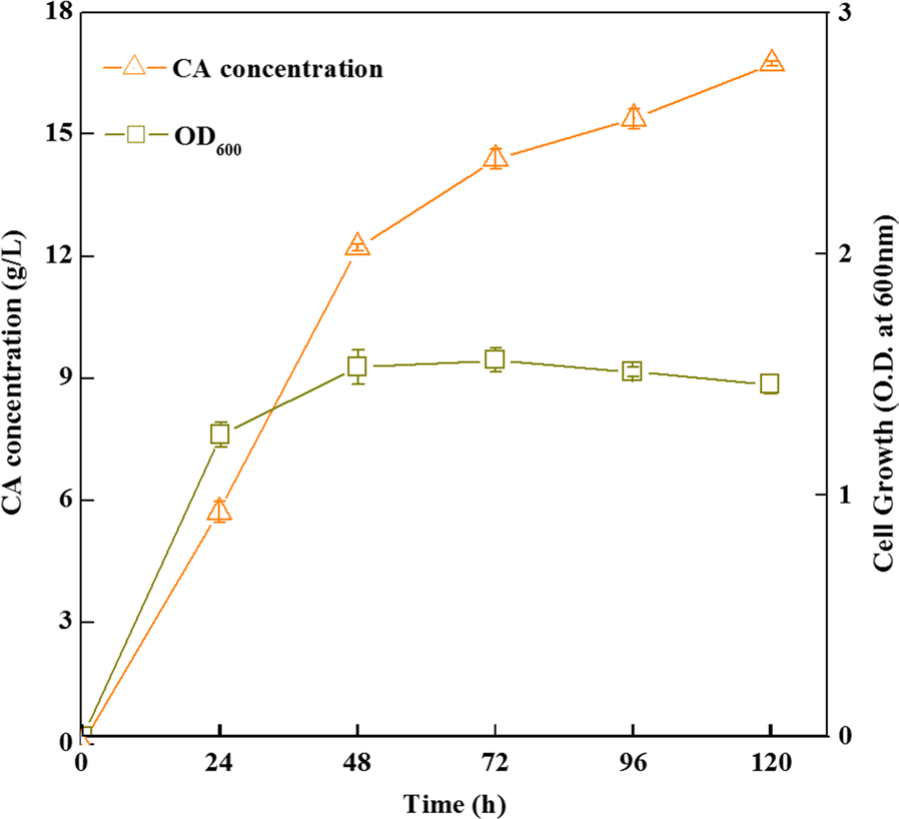
The growth of strain CPB6 and CA production under the optimized medium in a 5 L fermentation tank for 5-days cultivation. All data were presented as means ± standard deviations (n = 3)

The response surface plots in the Fig. 4 described the interactions between any two variables when other variables were run at their intermediate levels. The Fig. 4a illustrated that the CA yield increased with the increase of lactate and sucrose concentrations. Figure 4b showed that the effects of lactate and butyrate on CA production, and the CA yield increased with increasing lactate concentration when butyrate was below 16 g/L. Figure 4c demonstrated the positive effects of sucrose and butyrate on CA production. Finally, the optimal parameters for a maximum CA yield (16.27 g/L) were 13.30 g/L of sucrose, 22.35 g/L of lactate and 16.20 g/L of butyrate. An experiment was implemented in a 5 L fermentation tank to verify the optimal medium parameters resulted from Box–Behnken design, and 16.73 g/L CA production was obtained in our verification experiment with a maximum productivity rate of 6.5 g/L/day (Fig. 5). It was approximately equal to the predicted value. The perfect fit between the actual value and the theoretical value suggested the veracity of the model.

To our knowledge, *M*. elsdenii is reported to be the first pure culture capable of producing CA from lactate, but CA is only by-product (0.48 g/L) of propionate and butyrate [22]. The well-known ethanol-based CA—producing bacteria *Clostridium kluyveri* can produce 12.8 g/L (110 mM) CA from 23 g/L (500 mM) ethanol and 7.2 g/L (120 mM) acetate [10], which is the highest concentration of CA reported by pure culture from a synthetic substrate of ethanol and acetate. *Clostridium* sp. BS-1 can produce 6.96 g/L (60 mM) CA from galactitol (15 g/L) and butyrate (6.78 g/L) [28] and *Megasphaera* sp. MH can produce 9.7 g/L (83.5 mM) CA from 20 g/L fructose with 8.2 g/L (100 mM) acetate and 11 g/L (100 mM) butyrate [24]. Compared to other CA-producing strains, strain CPB6 showed the highest CA titer (16.73 g/L, 144 mM) and very high productivity (6.50 g/L/day) in a synthetic substrate containing lactate (22.35 g/L), butyrate (16.48 g/L) and sucrose (13.30 g/L). It is the maximum CA production for this strain in batch pure cultures. Notably, the maximum production rate increased roughly by 23% compared to previous study (6.50 vs 5.29 g/L/day), implying equivalent CA production would require less fermentation time in optimized medium. Overall, these results are very useful for large-scale CA production in future industrial application.

## Conclusions

Electronic acceptors and carbon sources are important for the growth of strain CPB6 and CA production. Acetate, butyrate and sucrose supplementation to the lactate-medium gave rise to significantly increased CA production. Exogenous propionate and pentanoate would result in the production of pentanoic acid and *n*-heptanoic acid. Butyrate and sucrose were the most appropriate electron acceptor and carbon source respectively for CA production from lactate by strain CPB6. An optimum medium composition containing 13.30 g/L sucrose, 22.35 g/L lactate and 16.48 g/L butyrate for CA production was acquired using RSM. Under such optimized conditions, CA production reached up to 16.73 g/L with a maximum productivity of 6.5 g/L/day.

## Methods

### Medium and cell culture

Strain CPB6 (GDMCC No.60133) was cultivated in the growth medium containing the following compounds per liter of distilled water: yeast extract 1.0 g, peptone 1.0 g, glucose 10 g, K_2_HPO_4_·3H_2_O 0.41 g, KH_2_PO_4_ 0.23 g, NH_4_CL 0.25 g, MgSO_4_ × 7H_2_O 0.20 g, trace element solution SL-10 [17] 1.0 mL, selenite-tungstate solution [17] 1.0 mL, NaHCO_3_ 2.5 g, l-cysteine 0.25 g, Na_2_S·9H_2_O 0.25 g and resazurin 0.5 mg. The oxygen was removed by flushing with nitrogen gas for 20 min (100 mL). The pH of the medium was adjusted to 5.5–5.8 using 6 M NaOH and 6 M HCL. After sterilization and cooling, 1 ml anoxic and filter-sterilized vitamin solution [17] was added. The strain CPB6 was incubated at 37 °C for 24 h as seed inoculum (OD_600_ = 0.8–1.0) for batch experiments. An inoculum concentration of 5% was used for each assay. All inoculated operations were performed in the AG300 anaerobic workstation (Gene Science, USA). The lactate-medium (12 g/L), replacing glucose by d, l-sodium lactate in the growth medium, was used for MCCAs production with different EAs by strain CPB6.

### Effect of EA on fermentation products

The effects of different EAs on cell growth and CA production were evaluated to screen the most suitable substrate (EA). Batch experiments were carried out in the lactate-medium supplemented with different EAs (final concentration of 100 mM except 50 mM caproate) including sodium acetate (8.2 g/L), sodium propionate (9.6 g/L), sodium butyrate (11 g/L), pentanoate (12.4 g/L), and sodium caproate (6.9 g/L), respectively. Additionally, the effect of acetate and butyrate as cosubstrates on CA production was also investigated by adding 50 mM sodium acetate and sodium butyrate simultaneously to the lactate-medium. All fermentation tests were performed in 250 mL anaerobic serum bottles containing 100 mL of fermentation medium and incubated at 37 °C in a rotating shaker with 50 rpm. Each treatment was performed in triplicates.

### Effect of carbon source on cell growth and CA production

The effects of carbon sources on cell growth were tested by supplementing with 10 g/L glucose, maltose, fructose and sucrose, respectively. In addition, batch experiments were performed to assess the effect of sucrose (10 g/L) on CA production in the lactate-medium without or with EA. Each experiment was performed in triplicates.

### Optimization of culture medium for CA production

RSM is an effective method in predicting the relationship between key factors from a multivariable system by providing optimized levels [29]. It has been widely used in culture condition optimization for increasing metabolites production by microbes [28, 30, 31]. The Box–Behnken experimental design in the RSM has been widely used in biofuels production optimization such as butanol and butyric acid [29, 31]. The statistical results in the Box–Behnken experimental design can be used to study the relationships among a number of independent variables and responses [32].

Experiments based on three-levels of three variables (sucrose, lactate, and butyrate) were carried out by the response surface methodology (RSM). Three levels of the three variables, low (− 1), middle (0), high (+ 1), and the codes were shown in Table 3. Each experiment was performed in triplicates. All the data analysis was completed in Design-Expert 8.0 software (stat-ease, MN, USA).

### Analytical methods

Cell growth was monitored by measuring OD_600_ using a TU-1810 UV/Vis Spectrophotometer (Beijing Puxi Instrument Co. Ltd.). C2–C6 carboxylic acid were analyzed by HPLC (Agilent 1260 Infinity, USA) equipped with a differential refraction detector (RID) and a Hi-Plex H column (300 × 6.5 mm). In brief, the samples were diluted 20 times with distilled water after centrifugation at 8000 rpm for 5 min, and then were filtrated by 0.22-μm filter (Millipore Corp, Bedford, MA). The resulting solution was injected into the HPLC. C7–C8 carboxylic acids were analyzed by 7890B gas chromatography (Agilent Technologies, USA) with a flame ionized detector (FID) and DB-FFAP (30 m × 0.53 mm × 1 um). The sample from the broth was adjusted to pH of 2 using 6 M HCL, and was extracted with ethyl acetate. The organic layer was filtrated through 0.22 μm filter (Millipore Corp, Bedford, MA) and 2 μL resulting solution was then injected into the gas chromatograph. The split ratio is 5:1; the injector temperature is 250; the detector temperature is 300; the column temperature is 100 °C for 5 min and ramps up to 210 °C at 10 °C/min for 12 min. Nitrogen (99.9999%) was used as the carrier gas at the rate of 3 mL/min; the hydrogen flow rate was 40 mL/min; the air mix flow rate was 400 mL/min.

## Additional file

**Additional file 1: Table S1.** Full factorial experimental design matrix and results. **Table S2.** Modle coefficients on CA production estimated by fractional factorial experimental design. **Table S3.** Steepest ascent experimental design matrix and results. **Table S4.** Analysis of variance (ANOVA) of the Box–Behnken design on CA production.

## Abbreviations

MCCA: medium-chain carboxylic acid
CA: *n*-caproic acid
EA: electron acceptor
SCCA: short-chain carboxylic acid
COD: chemical oxygen demand
RSM: response surface methodology
GC: gas chromatography
HPLC: high-performance liquid chromatography
