# Supplementary material for: Improvement of n-caproic acid production with Ruminococcaceae bacterium CPB6: selection of electron acceptors and carbon sources and optimization of the culture medium
Source: Microb Cell Fact. 2018 Jun 25;17:99. doi: 10.1186/s12934-018-0946-3 (PMC6019802; doi:10.1186/s12934-018-0946-3)
Supplement: Supplementary file 1 — Additional file 1: Table S1. Full factorial experimental design matrix and results. Table S2. Modle coefficients on CA production estimated by fractional factorial experimental design. Table S3. Steepest ascent experimental design matrix and results. Table S4. Analysis of variance (ANOVA) of the Box–Behnken design on CA production. [file 12934_2018_946_MOESM1_ESM.docx]

**Additional file**

**Table S1** Full factorial experimental design matrix and results

| Run | Yeast extract | Tryptone | Sucrose | Butyrate | Caproate (g/L) |
| --- | --- | --- | --- | --- | --- |
| 1 | 10.00 | 10.00 | 20.00 | 20.00 | 12.02 |
| 2 | 1.00 | 1.00 | 20.00 | 0.00 | 5.17 |
| 3 | 1.00 | 10.00 | 0.00 | 0.00 | 1.65 |
| 4 | 1.00 | 10.00 | 0.00 | 20.00 | 7.47 |
| 5 | 1.00 | 1.00 | 20.00 | 20.00 | 11.16 |
| 6 | 10.00 | 10.00 | 20.00 | 0.00 | 6.31 |
| 7 | 10.00 | 1.00 | 20.00 | 0.00 | 6.25 |
| 8 | 10.00 | 1.00 | 0.00 | 20.00 | 6.89 |
| 9 | 10.00 | 1.00 | 20.00 | 20.00 | 11.68 |
| 10 | 1.00 | 10.00 | 20.00 | 0.00 | 5.87 |
| 11 | 10.00 | 10.00 | 0.00 | 0.00 | 2.47 |
| 12 | 1.00 | 1.00 | 0.00 | 20.00 | 8.12 |
| 13 | 1.00 | 1.00 | 0.00 | 0.00 | 1.65 |
| 14 | 10.00 | 1.00 | 0.00 | 0.00 | 1.79 |
| 15 | 1.00 | 10.00 | 20.00 | 20.00 | 12.59 |
| 16 | 10.00 | 10.00 | 0.00 | 20.00 | 8.22 |

| **Table S2** Modle coefficients on CA production estimated by full factorial experimental design | | | | |
| --- | --- | --- | --- | --- |
| Source | Coefficient | Standard error | t-value | p-value |
| A-yeast extract | 0.12 | 0.052 | 7.21 | 0.0549 |
| B-tryptone | 0.24 | 0.052 | 28.71 | 0.0059 |
| C-sucrose | 2.05 | 0.052 | 2039.81 | <0.0001 |
| D-butyrate | 2.94 | 0.052 | 4189.07 | <0.001 |
| AC | 0.062 | 0.052 | 1.86 | 0.2444 |
| AD | -0.19 | 0.052 | 17.19 | 0.0143 |
| BC | 0.073 | 0.052 | 2.60 | 0.1824 |
| BD | 0.063 | 0.052 | 1.94 | 0.2366 |
| ABC | -0.27 | 0.052 | 36.56 | 0.0038 |
| BCD | 0.063 | 0.052 | 1.94 | 0.2366 |
| ABCD | -0.11 | 0.052 | 5.81 | 0.0735 |
| R^2^=0.9994, R2 adj=0.9976, R2 prep =0.9899, Adeq Precision=70.338 | | | | |

**Table S3** Steepest ascent experimental design matrix and results

| Run | Lactate | Sucrose | Butyrate | Caproate(g/L) |
| --- | --- | --- | --- | --- |
| 1 | 12 | 5 | 8 | 9.30 |
| 2 | 18 | 10 | 12 | 13.21 |
| 3 | 24 | 15 | 16 | 15.15 |
| 4 | 30 | 20 | 20 | 10.37 |
| 5 | 36 | 25 | 24 | 7.76 |
| 6 | 42 | 30 | 30 | 2.60 |

| **Table S4** Analysis of variance (ANOVA) of the Box-Behnken design on CA production^a^ | | | | | |
| --- | --- | --- | --- | --- | --- |
| Source | Statistics | | | | |
|  | Sum of squares | df | Mean square | F-Value | P-Value |
| Model | 12.14 | 9 | 1.35 | 47.28 | <0.0001 |
| X_1_ | 0.92 | 1 | 0.92 | 32.40 | 0.0007 |
| X_2_ | 3.95 | 1 | 3.95 | 138.33 | <0.0001 |
| X_3_ | 1.20 | 1 | 1.20 | 42.09 | 0.0003 |
| X_1_X_2_ | 0.044 | 1 | 0.044 | 1.55 | 0.2539 |
| X_1_X_3_ | 0.35 | 1 | 0.35 | 12.20 | 0.0101 |
| X_2_X_3_ | 0.12 | 1 | 0.12 | 4.05 | 0.084 |
| X2 1 | 2.18 | 1 | 2.18 | 76.27 | <0.0001 |
| X2 2 | 2.52 | 1 | 2.52 | 88.38 | <0.0001 |
| X2 3 | 0.36 | 1 | 0.36 | 12.75 | 0.0091 |
| Residual | 0.20 | 7 | 0.029 |  |  |
| Lack of Fit | 0.084 | 3 | 0.028 | 0.97 | 0.4896 |
| Pure Error | 0.12 | 4 | 0.029 |  |  |
| Cor Total | 12.34 | 16 |  |  |  |
| R^2^=0.9838, R2 adj=0.9630, R2 prep=0.8763, Adeq Precision=20.379 | | | | | |

^a^ x_1_, sucrose; x_2_, lactate; x_3_, butyrate.
